# 

**Published:** 1895-05

**Authors:** 


### CONTENTS.

ORIGINAL ARTICLES—

A Duty of the State Medical Association. By F. M. Ridley, M. D., LaGrange, Ga . . . . . 239

The Management of Hemorrhage After Tonsil Operations. By A. W. Calhoun, M. D., LL. D., Atlanta, Ga. . . . . 245

Some Rare Cases in Obstetric Practice, With Special Reference to the Treatment. By C. H. Richardson, M. D., Montezuma, Ga. 247

CASE I.—Passage of Hard Non-Fusible Phosphate of Lime Calculus from Bladder. CASE II.—Polyuria Due to Tubercular Peritonitis. By William S. Gordon, M. D., Richmond, Va . . . 252

SOCIETY REPORTS—

Medical Association of Georgia........................................ 255

SELECTIONS AND ABSTRACTS—

Symptomatic Treatment of Influenza.............................. 272
Paralysis in the Course of Pneumonia.............................. 274
Death from Antitoxin.............................................. 275
College Graduates................................................. 276

BOOK REVIEWS..........................................................277-278

EDITORIAL—

Dr. Frank Morris Ridley........................................... 279
Medical Meetings.................................................. 279
Editorial Notes................................................... 282
Special Notes..................................................... 284
Prescriptions..................................................... 288

